# Identification of Houge type of X-linked syndromic mental retardation caused by *CNKSR2* truncated variants

**DOI:** 10.1186/s13052-025-01877-0

**Published:** 2025-02-07

**Authors:** Si-Hua Chang, Jie-Yuan Jin, Yi-Qiao Hu, Run-Yan Wang, Rong Xiang, Xia Wang

**Affiliations:** 1 Department of Pediatrics, Xiangya Hospital, Central South University, No.78 Xiangya Road, Changsha, 410000 China; 2 Department of Hand and Microsurgery, Xiangya Hospital, Central South University, Changsha, 410000 China; 3 School of Life Sciences, Central South University, No.172 Tongzipo Road, Changsha, 410000 China; 4 National Clinical Research Center for Geriatric Disorders, Xiangya Hospital, Central South University, Changsha, 410000 China; 5 Microsurgery & Reconstruction Research Center, Xiangya Hospital, Central South University, Changsha, 410000 China

**Keywords:** CNKSR2, MRXSHG, Minigene, Truncated variants, Mental retardation

## Abstract

**Background:**

Houge type of X-linked syndromic mental retardation (MRXSHG) is a type of X-linked condition which is mainly manifested as delayed development, mental retardation, epilepsy that begins at an early age, and delayed language acquisition. MRXSHG is a serious disorder with *CNKSR2* variant and at least 34 variants have been identified in MRXSHG patients. However, the genotype-phenotype correlation and variants characteristics of *CNKSR2* need further investigation and improvement.

**Methods:**

Two Chinese MRXSHG families were recruited, and their genetic causes were investigated using whole-exome sequencing (WES), Sanger sequencing, and bioinformatics analysis. To verify the impact of these variants, we used real-time PCR and minigenes consisting of exon 14, intron 14, and exon 15 from both the wild-type and the c.1658-3_1676del DNA sequences.

**Results:**

In this study, we reported two Chinese boys with MRXSHG and described some rare MRXSHG phenotypes, such as delayed bone age, slightly widened right fissure, and an underdeveloped right temporal lobe, characterized by reduced growth and volume compared to typical development. Two novel variants in *CNKSR2* (c.1658-3_1676del and c.1102G > T, p.Gly368*) were identified in these cases. Minigenes results revealed that variant c.1658-3_1676del produced an aberrant spliceosome assembly.

**Conclusions:**

We identified two novel *CNKSR2* variants in MRXSHG families, expanding the variant spectrum of *CNKSR2*, enriching MRXSHG-related phenotypes, and contributing to genetic counseling for MRXSHG patients.

**Supplementary Information:**

The online version contains supplementary material available at 10.1186/s13052-025-01877-0.

## Background

The *CNKSR2* gene, located on the X chromosome at Xp22.12, comprises 22 exons and encodes the connector enhancer of kinase suppressor of Ras 2 (CNKSR2). This protein functions as a scaffold and is highly expressed in the brain [1], playing a crucial role in mediating numerous signaling pathways and neuronal morphogenesis [2–5]. The initial identification of a pathogenic *CNKSR2* variant in a boy with neurodevelopmental disorders was reported by Houge et al. in 2012 [6]. Consequently, CNKSR2-related disorders were named the “Houge type of X-linked syndromic mental retardation (MRXHG, MIM 301008)”. Whereas the genotype-phenotype correlation and variants characteristics of *CNKSR2* need further investigation and improvement.

MRXSHG is a type of mental retardation characterized by X-linked inheritance. Affected individuals often present with development delays, mental retardation, seizure with an onset at early ages, and delayed language acquisition. Additionally, symptoms frequently include inattentiveness, hyperactivity, and common sleep disturbances [7–9]. Electroencephalographic (EEG) studies in MRXSHG cases typically show distinctive features such as continuous spike and wave during slow sleep (CSWS) or centrotemporal activity [10]. Notably, severely affected patients are those with hemizygous variants. In contrast, heterozygous females usually exhibit no symptoms or only mild phenotypes, likely due to X-inactivation [11].

In this report, we reported two Chinese boys with MRXSHG and descripted some rare MRXSHG phenotypes. Two novel variants in *CNKSR2* (c.1658-3_1676del and c.1102G > T, p.Gly368*) were identified in these two cases by whole-exome sequencing (WES), which were determined to be underlying genetic causes in their families. The deletion variant c.1658-3_1676del, spanning the splice junction between Intron 14 and Exon 15, prompted us to construct minigenes that revealed an aberrant spliceosome assembly due to this deletion. Our discovery not only broadens the range of known *CNKSR2* variants and enriches MRXSHG-related phenotypes but also offers significant insights for the molecular diagnosis and genetic counseling in MRXSHG patients. Furthermore, they pave the way for further research into the pathogenesis of MRXSHG associated with *CNKSR2* variants.

## Methods

### Subjects

The research protocol was approved by the Review Board of the Xiangya Hospital of Central South University in China (2023111889) and informed consent was obtained from all participants. Two unrelated family members participated in this study. Subjects provided their blood and consented to publish the clinical details.

### Whole-exome sequencing

We extracted their genomic DNA from the peripheral blood of subjects with the DNeasy Blood & Tissue Kit (Qiagen, Valencia, USA). Berry Genomics Company Limited (Chengdu, China) provided whole exomes sequencing services including exome capture and high-throughput sequencing. They also did some essential bioinformatic analysis such as variant identification, standard filtering, and annotation by MutationTaster (http://www.mutationtaster.org), PolyPhen-2 (http://genetics.bwh.harvard.edu/pph2), SIFT (http://provean.jcvi.org/index.php), GERP (http://mendel.stanford.edu/sidowlab/downloads/gerp/index.html), CADD (https://cadd.gs.washington.edu/snv), GnomAD (http://gnomad.broadinstitule.org), 1000G (https://www.internationalgenome.org), and OMIM (https://www.omim.org).

### Variant validation

We used Sanger sequencing to validate the variants after the common filtering. Integrated DNA Technologies (https://sg.idtdna.com/Primerquest/Home/Index) helped us to design the primer pairs (Case 1 F: CTCGGCCATTTACTACCCTAAC; Case 1 R: GGTGCTTTCTCGTCTTCCTT; Case 2 F: GTGTGTCACTTATGTGAGGGAA; Case 2 R: TGCATCCTTTAGGACAAACCA) which were used for PCR amplification. The PCR products were determined by the ABI 3100 Genetic Analyzer (Thermo Fisher Scientific, Waltham, USA).

### Mutant protein modeling

We download CNKSR2 protein structure information from AlphaFold Protein Structure Database (https://alphafold.ebi.ac.uk/entry/Q8WXI2). We used PyMol to construct mutant CNKSR2 models based on the wild-type structure.

### Minigene assay

We procured plasmids harboring both wild-type and c.1658-3_1676del DNA sequences corresponding to Exon 14, Intron 14, and Exon 15 within the pcDNA 3.1(+) vector from Sangon Biotech (Shanghai, China). Sanger sequencing was performed. Wild-type and mutant plasmids were transfected into HEK293 cells using Lipofectamine 3000 (Thermo Fisher Scientific, Waltham, MA, USA). After a 36-hour incubation period post-transfection, total RNA extraction was carried out utilizing the MolPure Cell RNA Kit (Yeasen Biotech Co., Ltd., Shanghai, China). The isolated RNA was then subjected to reverse transcription using the All-in-One First-Strand Synthesis MasterMix (with dsDNase) Kit (Best Enzymes Biotech Co., Ltd., Jiangsu, China) to generate complementary DNA (cDNA). The cDNA sequences of recombined plasmids were amplified by RT-PCR and the primer pairs (F: CACCACCACGACTACAAAGA; R: TGCATCCTTTAGGACAAACCA) were designed by Integrated DNA Technologies (https://sg.idtdna.com/Primerquest/Home/Index). PCR products were analyzed through electrophoresis on a 1% agarose gel.

### *Real-time PCR* (RT-PCR)

After removing of red blood cells via peripheral blood of subjects centrifugation, total RNA was extracted using the EASYspin RNA Mini Kit (Aidlab, RN07). The cDNA was synthesized using the All-in-One First-Strand Synthesis MasterMix (with dsDNase) (Yugong Biotech, Jiangsu, China). The RT-PCR experiments were performed using a Q9600 Series Real-Time PCR System (BIO-GENER, China). GAPDH was used as the reference gene. Primer pairs sequences were designed by Integrated DNA Technologies (GAPDH F: GGTGTGAACCATGAGAAGTATGA; GAPDH R: GAGTCCTTCCACGATACCAAAG; CNKSR2 F: GGAGTCTGTGACCACATCATATC; CNKSR2 R: GCCCTTCGCTTGGTTTAATG).

## Results

### Case description

**Fig. 1:**
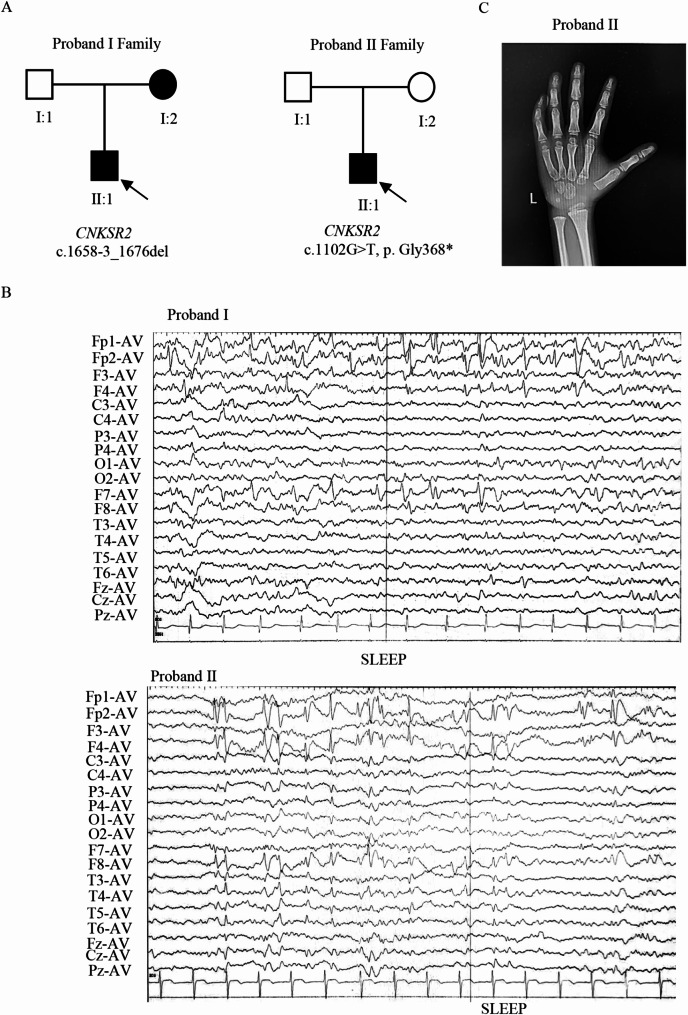
Pedigree, symptom, and EEG recording of the probands. (**A**) Family pedigree of *CNKSR2* variation cases (**B**) EEG results showed the presence of spike and waves. (**C**) 6 years old Proband II’s carpal bones were 4 years old and 6 months old, phalanges were 6 years old and 8 months old

Proband I (Fig. 1A), a 9-year-old boy from Hunan Province, China, exhibited global developmental delays. He has been diagnosed with epilepsy, delayed language, motor development, and has remained non-verbal since birth. The boy suffered from seizures with an onset at 3 years old. He has not been taking medication and has intermittent seizures. At 9 years of age, he had about three seizures. EEG analyses indicated normal background activity with numerous spikes and waves in the bilateral rolandic areas, prominently on the right side (Fig. 1B). Additional diagnostic reports from other hospitals noted a slightly wider right fissure and a marginally smaller right temporal lobe compared to the left. Tracing back her family history, his mother’s (I:2) exhibited below-normal intelligence levels (Table 1), while his father (I:1) showed no anomalies.

**Table 1:** Phenotypes of the affected in these families

Patient	Proband I	Proband II	Proband I Mother
***CNKSR2*** **variant**	c.1658-3_1676del	c.1102G > T, p.Gly368*	Unknown
**Age**	9	6	35
**EEG**	CSWS*	CSWS*	Unknown
**MRI**	The right fissure was slightly wider than the left and the right temporal lobe was slightly underdeveloped	Normal	Unknown
**Epilepsy**	Onset at age 3	Onset at age 4	None
**Speech delay**	No speech	Language expression and comprehension were below average and speech was slurred	None
**Cognition and behavior**	Motor development delay	Severe delays in adaptive functioning, fine motor skills	Intelligence is lower than normal

*CSWS: continuous spike and slow wave

Proband II (Fig. 1A) was a 6-year-old boy. At the age of four, the patient began to have recurrent fevers and seizures, each episode lasting about 1–2 min. His language skills were below average and he exhibited slurred speech. A Gesell developmental assessment categorized his retardation as mild to moderate. EEG recordings during sleep revealed spikes and waves in the bilateral polus frontalis, frontal and right middle posterior temporal areas (Fig. 1B). In 2019, he was diagnosed with inflammation of the left ear and adenoid hypertrophy. In the same year, the five-year-old boy had a fever lasting half a day and two generalized tonic-clonic seizures with loss of consciousness, each of which resolved spontaneously after about one minute. A 2020 reassessment at the hospital indicated severe delays in adaptive functioning, fine motor skills, and language, along with moderate delays in personal-social and gross motor skills. The Child Behavior Scale gave a total score of 23, indicating abnormal behavior (Table 2). We also assessed the patient’s overall intelligence using the WPPSI-IV, which includes the full scale IQ (FSIQ) and five indices: verbal comprehension index, visuospatial index, fluid reasoning index, working memory index, and processing speed index. The analysis yielded six scores in total. The FSIQ was 58, indicating an extremely low intelligence level, and all five indices were also below average (Table 3, Supplementary Material). In addition, the patient showed delayed bone age (Fig. 1C). Notably, the family history of the patient was unremarkable. Furthermore, it should be added that these two patients are unrelated.

**Table 2:** Gesell Developmental Schedules score in Proband II

Project	Developmental Quotient	Developmental Age (months)	Evaluation
Adaptability	34.4	27.4	Severe developmental delay
Big movements	45.1	36	Moderate developmental delay
Fine movements	36.4	29	Severe developmental delay
Language	39.8	31.7	Severe developmental delay
Personal-social	45.6	36. 4	Moderate developmental delay

**Table 3:** WPPSI-IV scaled score Sum to Composite score Conversion table

Scale	Scaled Scores	Composite Scores	Percentile Ranks	Confidence Intervals (95%)
Verbal Comprehension Index (VCI)	4	59	0.3	55–69
Visual Spatial Index (VSI)	7	64	1	59–77
Fluid Reasoning Index (FRI)	9	69	2	64–78
Working Memory Index (WMI)	8	67	1	62–77
Processing Speed Index (PSI)	11	75	5	69–88
Full Scale IQ (FSIQ)	18	58	0.3	54–65

### Genetic analysis

**Fig. 2:**
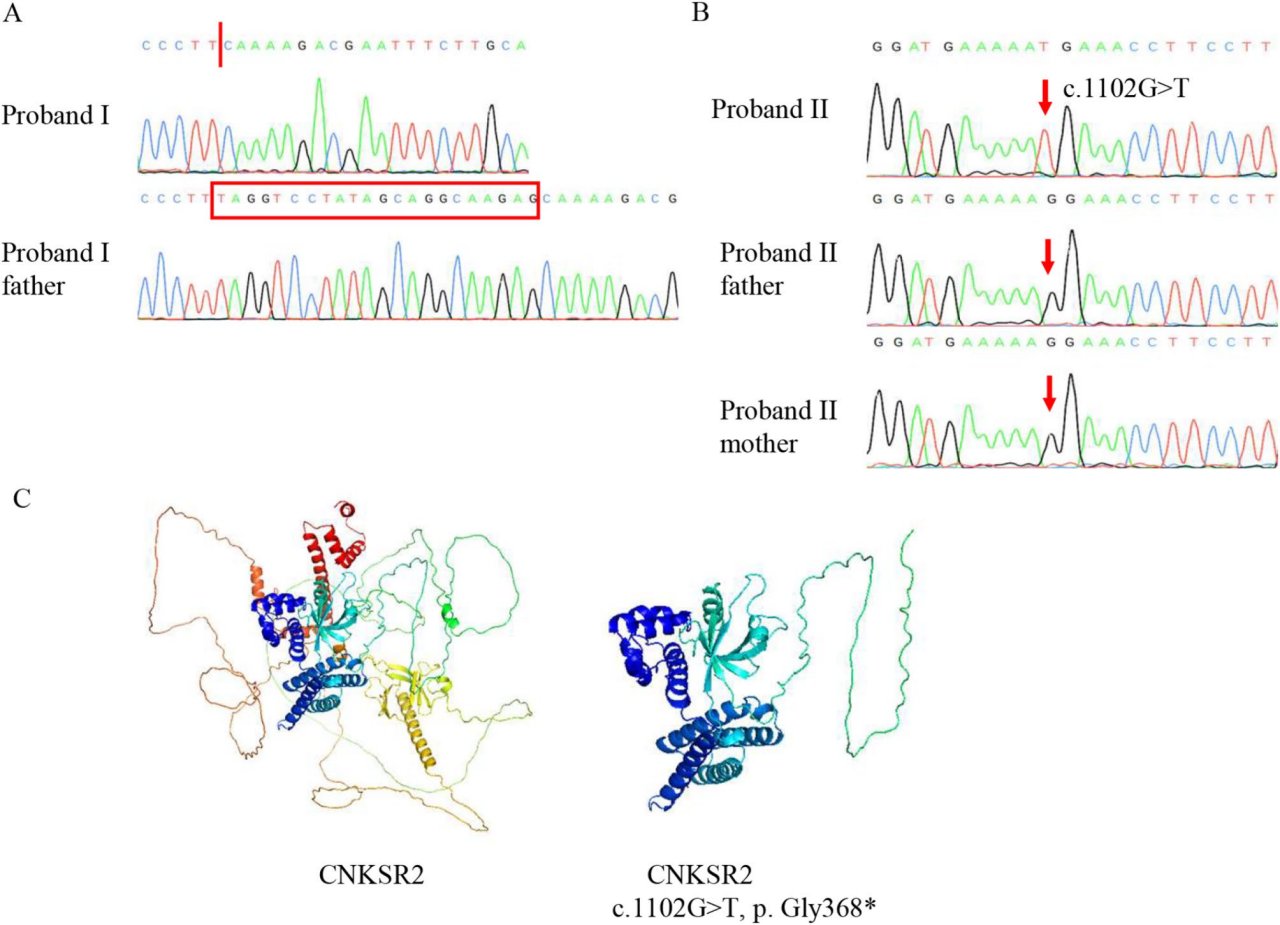
Sanger sequencing and protein modeling. (**A**) Sequencing results of the *CNKSR2* variant of Proband I and Proband I father using Sanger sequencing. Red arrow points the variant site. (**B**) Sequencing results of the *CNKSR2* variant of Proband II, Proband II mother and Proband II father using Sanger sequencing. Red arrow points the variant site. (**C**) Three-dimension model of CNKSR2 with wild type or variants

Exome sequencing was performed on genomic DNA isolated from peripheral blood samples of the probands to identify potential variants in these boys. We eliminated the variants with a frequency over 0.001 in GnomAD and below 15 in CADD, and the benign variants predicted by MutationTaster, Polyphen-2, and SIFT. Moreover, we prioritized variants that were nonsynonymous in exon regions or located at canonical splice acceptor or donor site. These remaining variants were matched against the genes associated with mental retardation and screened out through guidelines of the American College of Medical Genetics and Genomics (ACMG) to find the “Pathogenic” or “Likely pathogenic” gene. Finally, two potentially pathogenic variants in *CNKSR2* were filtered (NM_014927.5: c.1658-3_1676del and c.1102G > T, p.Gly368*; Table 4). We did Sanger sequencing of the potential causative variants for probands, and confirmed the variants for each family (case 1: c.1658-3_1676del and case 2: c.1102G > T, p.Gly368*; Fig. 2A).

**Table 4:** Variants identified in the proband by whole-exome sequencing

patient	Gene	Variant	Pathogenicity prediction	GnomAD	1000G	OMIM clinical phenotype	American College of Medical Genetics classification
Proband I	*CNKSR2*	NM_014927.5: c.1658-3_1676del	MutationTaster: D* Polyphen-2: - SIFT: - CADD: - GERP: -	-	-	XL, Intellectual developmental disorder, X-linked syndromic, Houge type.	Pathogenic (PVS1, PM2, PP3)
Proband II	*CNKSR2*	NM_014927.5: c.1102G > T, p.Gly368*	MutationTaster: D* Polyphen-2: - SIFT: - CADD: 39 GERP: C	-	-		Pathogenic (PVS1, PS2, PM2, PP3)

*D: disease causing; C: conservative; XL: X-linked inheritance

In accordance with the standards and guidelines of ACMG, we classified this *CNKSR2* variant (NM_014927.5: c.1658-3_1676del) as “Pathogenic”: [1] Most known *CNKSR2* variants are loss-of-function, and the variant of Proband I is a canonical splice site variant in *CNKSR2* (PVS1); [2] It is absent from controls in both the 1000 Genomes Project and GnomAD databases (PM2); [3] Multiple bioinformatics software predicted that this variant was pathogenic (PP3). For the other hand, we determined this *CNKSR2* variant of Proband II (NM_014927.5: c.1102G > T, p.Gly368*) as “Pathogenic”: [1] Similar to c.1658-3_1676del, c.1102G > T also was a truncated variant causing multi-exon deletion (PVS1); [2] It was assumed *de novo* with confirmation of paternity and maternity (PS2); [3] It also was not identified in controls (PM2); [4] This variant was predicted to be disease-causing by MutationTaster, PolyPhen-2, SIFT, et al. (PP3). To highlight the influence of the nonsense variant of *CNKSR2*, we used PyMoL for structural prediction. It was observed that the spatial structures of CNKSR2 proteins are significantly different between the wild-type and the patients (Fig. 2B).

**Fig. 3:**
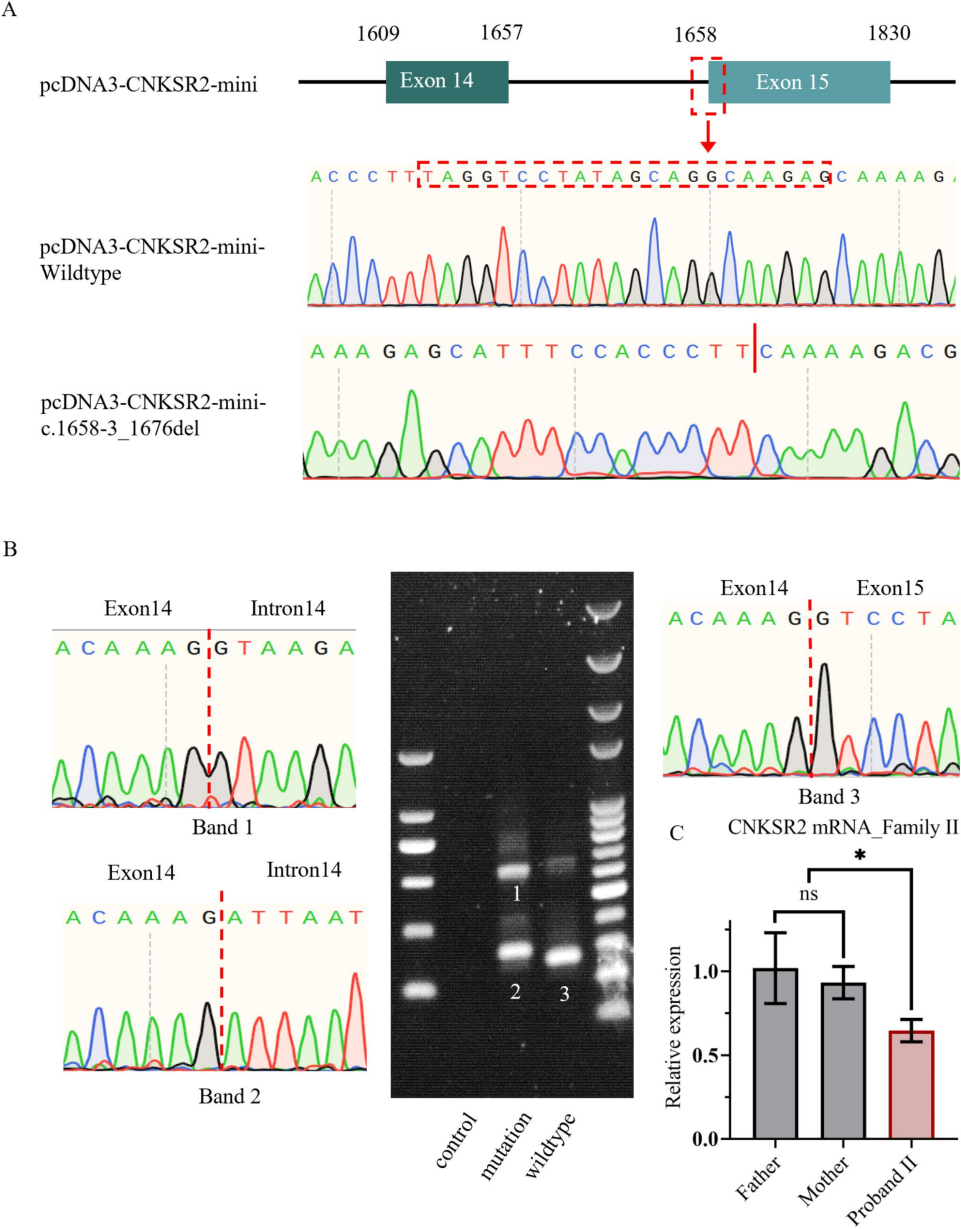
Minigene assay results of the*CNKSR2* c.1658-3_1676del variant and RT-PCR result of *CNKSR2* c.1102G > T (p.Gly368*) variant. (**A**) Construction of minigene wildtype and mutation plasmids. (**B**) Electrophoretic analysis depicting amplification products from cells expressing control, wild-type, and mutant minigene plasmids, along with sequencing chromatograms of the amplification products from cells expressing mutant plasmid (Band1 and Band2) and wildtype plasmid (Band3). (**C**) The expression levels of *CNKSR2* in the subjects were measured using real-time PCR

We utilized a minigene approach to further validate the pathogenicity of the variant c.1658-3_1676del identified in Family I (Fig. 3A). Agarose gel electrophoresis results demonstrated that the wild-type plasmid produced a single bright band of the expected size (276 bp) (Band 3), while the c.1658-3_1676del plasmid exhibited multiple bright bands (Band 1 and Band 2) that were different in size from the wild-type band (Fig. 3B). Sanger sequencing was conducted on these bands, and the results are depicted in Fig. 3. Sanger sequencing of these bands revealed that Band 1 included Exon 14, Intron 14, and Exon 15, representing the mutant minigene itself. Mutant Band 2, in addition to Exon 14 and 15, contained a small portion of Intron 14. (Fig. 3B). The results indicated that after the variant site at the splicing site, novel non-classical splicing sites were generated. Furthermore, due to inefficient splicing, numerous un-spliced products were simultaneously produced. In Family II, real-time PCR was performed to assess the pathogenicity of the c.1102G > T (p.Gly368*) variant (Fig. 3C). The results demonstrated a significant reduction in RNA levels compared to the wild-type, suggesting that the mutant transcript undergoes nonsense-mediated mRNA decay.

## Discussion

In this study, we report on two boys carrying the *CNKSR2* variants c.1658-3_1676del or c.1102G > T. Minigene analysis showed that variant c.1658-3_1676del caused the alteration of the original splice site, resulting in a frameshift. Although the Minigene results may not fully reflect the in vivo conditions, further validation is constrained by the inability to perform RNA sequencing, as Family I is currently out of the province and unwilling to continue providing fresh blood samples. These two boys both had mental retardation, developmental delay, and early-onset epilepsy, consistent with past reports of pathogenic variants in *CNKSR2* [10, 12, 13]. Previous studies found that in most patients with MRXSHG, the predominant topography of sleep-related EEG epileptic discharges was primarily frontal, with potential spreading to the central or temporal regions, suggesting that the epileptic activity primarily affects the frontal areas, particularly during sleep [10]. The presence of spikes and waves on EEG had been documented in both boys.

As other MRXSHG cases, both boys in our cases also had speech and language delays, one unable to speak at all and the other with a lisp. This was due to the selective and widespread expression of *CNKSR2* in various brain regions, including cerebellum and nucleus caudatus, which are involved in speech production [14, 15]. Due to X inactivation, most female carriers of the variant typically exhibit no significant phenotype or only mild mental retardation, as observed in the mother of Proband I who demonstrated lower intelligence. In addition, in many previous reports, the vast majority of patients with CNKSR-related disorders had normal MRI results [10, 12], some had abnormal hypoplasia of corpus callosum, hydrocephalus or widened bilateral ventricles [13, 16]. In our cases, MRI results indicated a slightly widened right fissure and an underdeveloped right temporal lobe, and X ray showed delayed bone age, findings that have not been previously reported. Our reports widen our understanding of the genotype-phenotype correlation of *CNKSR2* variants.

**Fig. 4:**
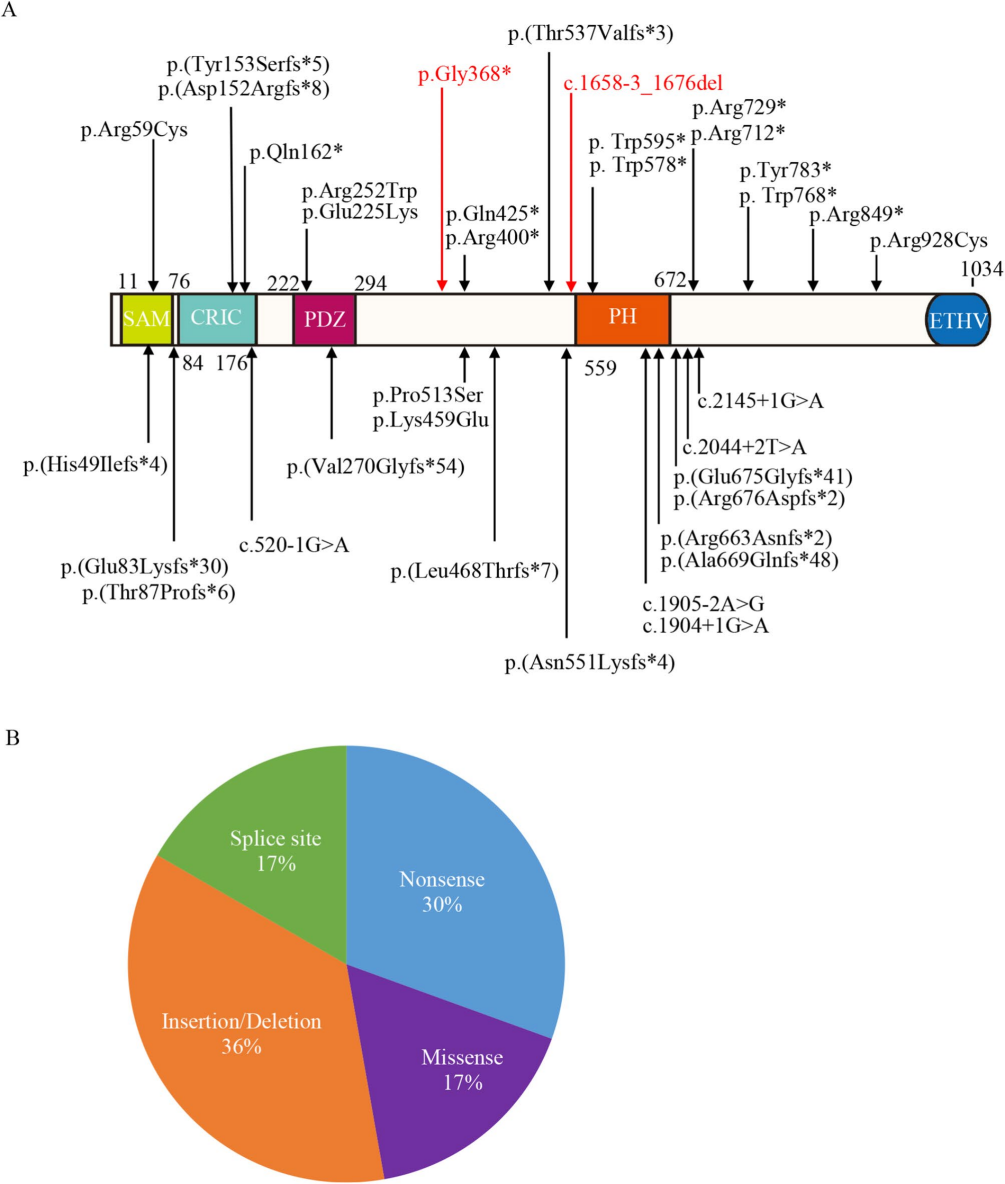
Pathogenic variants were reported in literature and this report. (**A**) 36 variants were reported in literature and report. (**B**) The proportion of nonsense, splice site, missense and insertion/deletion

CNKSR2 isoform 1 contains five different modules: one sterile alpha motif (SAM) domain, one conserved region in CNKSR (CRIC) domain, one PSD-95/Dlg-A/ZO-1 (PDZ) domain, one Pleckstrin homology (PH) domain and one C-terminal ETHV motif. These domains function as a protein-protein interacting domain and participate in different signaling machineries [11]. In this study, we identified a nonsense variant and a splice site variant in *CNKSR2*, predicted to produce a truncated protein and a frameshift mutant impacting the PH domain and C-terminal ETHV motif. (Fig. 4A). PH domains, typically consisting of approximately 120 amino acids, are small protein modules that can bind membrane phosphatidylinositol, commonly found in various proteins associated with cellular signaling, cytoskeletal rearrangement, and other important biological processes [17]. It is also reported to stimulate the MAPK pathway and help CNKSR2 locate at synapses [18, 19]. The identified variants may lead to truncated or frameshift proteins, potentially disrupting specific CNKSR2 functions, such as affecting some downstream signaling pathways like Ral small GTPase pathways, but the specific mechanisms need to be further studied.

As far, at least 34 variants (excluding genomic deletions) have been reported including nonsense variants, splice site variants, and small insertions and/or deletions (Fig. 4A). A significant proportion of these variants are truncating mutations (Fig. 4B). It suggests that loss-of-function variants of *CNKSR2* represent the primary molecular mechanism underlying the pathogenesis of MRXSHG, with the disruption or absence of CNKSR2’s normal functions playing a pivotal role. Many specific genes and variations in specific genomic regions significantly affect clinical phenotypes, highlighting the importance of genotype-phenotype correlation analysis in the diagnosis and research of genetic diseases [20–26]. Our review exhibited the characteristic of *CNKSR2* variants to facilitate the genetic counseling and molecular diagnostics for MRXSHG.

## Conclusion

Generally, our study reports two novel *CNKSR2* variants (c.1658-3_1676del and c.1102G > T, p.Gly368*), which were identified in two boys with MRXSHG by using of WES and Sanger sequencing and determined as pathogenic variants based on ACMG guidance and standard. Minigene assays confirmed that the c.1658-3_1676del variant alters splicing. Our description enriched the phenotype profile of *CNKSR2*-related neurodevelopmental and epilepsy disorders, extended the pathogenic spectrum of *CNKSR2* variants, and brought us closer to the goal of serving as a reference in genetic consultation, prenatal diagnosis and investigating treatment options for patients with MRXSHG.

## Electronic supplementary material

Below is the link to the electronic supplementary material.

Supplementary Material 1

## Data Availability

The datasets used and/or analysed during the current study are available from the corresponding author on reasonable request.

## Abbreviations

MRXSHG: Houge type of X-linked syndromic mental retardation
WES: Whole-exome sequencing
ACMG: American college of medical genetics and genomics
EEG: Electroencephalographic
CNKSR2: Connector enhancer of kinase suppressor of ras 2
IQ: Intelligence quotient
RT-PCR: Real-time PCR
