# Supplementary material for: Identification of Houge type of X-linked syndromic mental retardation caused by CNKSR2 truncated variants
Source: Ital J Pediatr. 2025 Feb 7;51:31. doi: 10.1186/s13052-025-01877-0 (PMC11806549; doi:10.1186/s13052-025-01877-0)
Supplement: Supplementary file 1 — Supplementary Material 1 [file 13052_2025_1877_MOESM1_ESM.pdf]

WPPSI™-IV

WECHSLER PRESCHOOL AND PRIMARY SCALE OF INTELLIGENCE — FOURTH EDITION

Summary Page

Applicable age **4:0～6:11**

Number: 0007628004

Name:

Doctor:

	year	month	day
Data	2020	06	28
Birth	2013	11	05
Age	6	7	23

Raw score and scaled score

Indexes	Score	Scaled score (Verbal Comprehension)	Scaled score (Visual Spatial)	Scaled score (Fluid Reasoning)	Scaled score (Working Memory)	Scaled score (Processing Speed)	Scaled score (Full Scale)	Scaled score
Block Design (BD)	8		3				3	
Information (IN)	10	1					1	
Matrix Reasoning (MR)	3			3			3	
Bug Search (BS)	10					4	4	
Picture Memory (PM)	6				4		4	
Similarities (SI)	4	3					3	
Picture Concepts (PC)	7			6			(6)	
Cancellation (CA)	25					7	(7)	
Zoo Locations (ZL)	6				4		(4)	
Object Assembly (OA)	9		4				(4)	
Animal Coding (AC)	10						(5)	
Receptive Vocabulary (RV)	12							2
Picture Naming (PN)	8							2
Random Cancellation (CAR)	15							9
Sequential Cancellation (CAS)	10							6
Total		4	7	9	8	11	18	

Scaled score profile of indexes

	Verbal Comprehension		Visual Spatial		Fluid Reasoning		Working Memory		Processing Speed		
	IN	SI	BD	OA	MR	PC	PM	ZL	BS	CA	AC
Scaled score	1	3	3	4	3	6	4	4	4	7	5

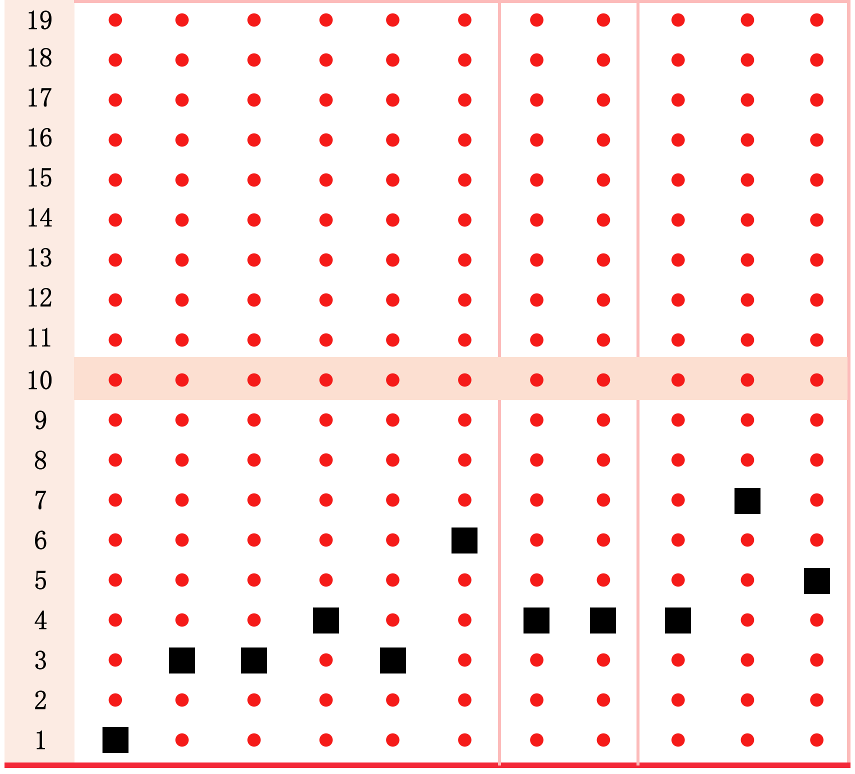

Scaled Score Sum to Composite Score Conversion Table.

Scaled	Scaled score	Composite score		Percentile ranks	Confidence interval 95%
Verbal Comprehension	4	VCI:	59	0.3	55-69
Visual Spatial	7	VSI:	64	1	59-77
Fluid Reasoning	9	FRI:	69	2	64-78
Working Memory	8	WMI:	67	1	62-77
Processing Speed	11	PSI:	75	5	69-88
Full Scale IQ	18	FSIQ:	58	0.3	54-65

Scaled score profile

CV	VS	FR	WM	PS	FSIQ
59	64	69	67	75	58

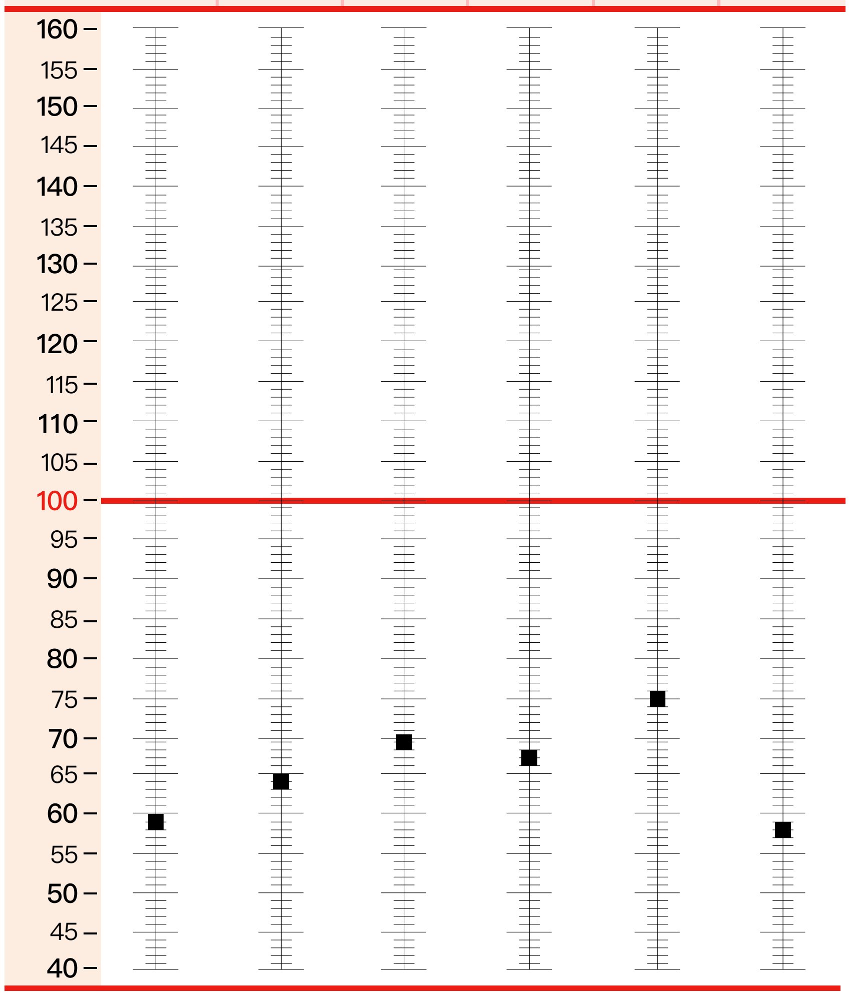

Remarks:

PEARSON

京美心测

《WPPSI-IV 中文版》经 NCS Pearson, Inc. 授权，由珠海市京美心理测量技术开发有限公司翻译，改编和印制。

版权所有，翻版必究。

WPPSI-IV
WECHSLER PRESCHOOL AND PRIMARY SCALE OF INTELLIGENCE — FOURTH EDITION

韦氏幼儿智力量表第四版中文版

记分册. 汇总页

适用年龄 4:0～6:11

个案编号: 0007628004
儿童姓名: 李宇晨
主试姓名: 段浩林

计算儿童年龄

	年	月	日
测试日期	2020	06	28
出生日期	2013	11	05
实足年龄	6	7	23

原始分数与量表分数转换表

分测验		原始分数	量表分数						
积木	BD	8		3				3	
常识	IN	10	1					1	
矩阵推理	MR	3			3			3	
找虫	BS	10					4	4	
图片记忆	PM	6				4		4	
类同	SI	4	3					3	
图画概念	PC	7			6			(6)	
划消	CA	25					7	(7)	
动物家园	ZL	6				4		(4)	
拼图	OA	9		4				(4)	
动物译码	AC	10						(5)	
指认图片	RV	12							2
图片命名	PN	8							2
随机划消	CAR	15							9
有序划消	CAS	10							6
量表分数总和			4	7	9	8	11	18	
			言语理解	视觉空间	流体推理	工作记忆	加工速度	全量表	

分测验量表分数剖析图

言语理解		视觉空间		流体推理		工作记忆		加工速度		
IN	SI	BD	OA	MR	PC	PM	ZL	BS	CA	AC
1	3	3	4	3	6	4	4	4	7	5

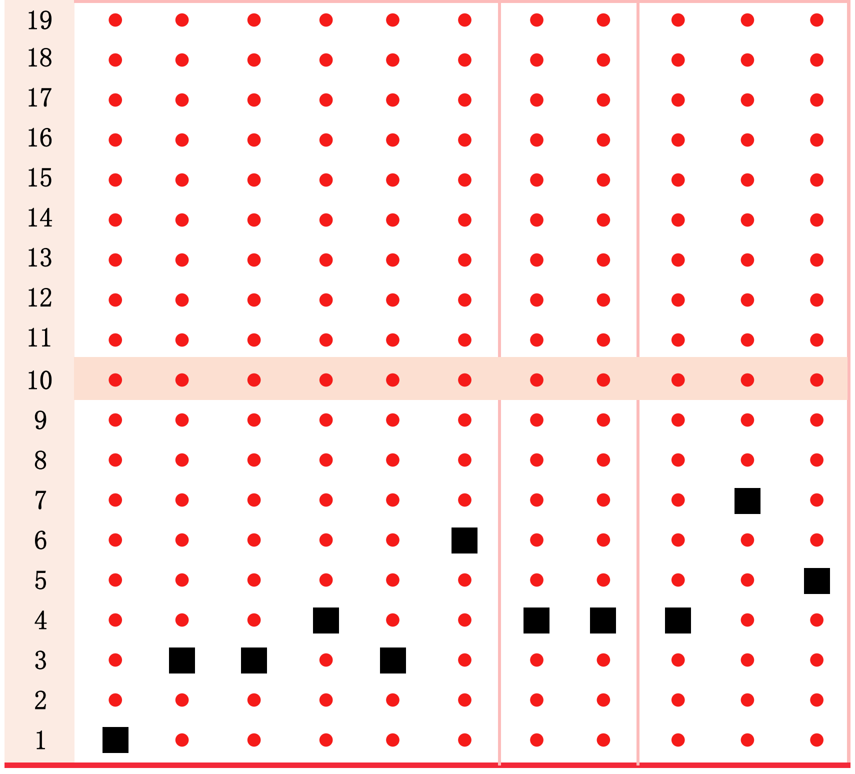

量表分数总和与合成分数转换表

量表	量表分数总和	合成分数		百分等级	置信区间 95%
言语理解	4	VCI:	59	0.3	55-69
视觉空间	7	VSI:	64	1	59-77
流体推理	9	FRI:	69	2	64-78
工作记忆	8	WMI:	67	1	62-77
加工速度	11	PSI:	75	5	69-88
全量表	18	FSIQ:	58	0.3	54-65

合成分数剖析图

言语理解	视觉空间	流体推理	工作记忆	加工速度	总智商
59	64	69	67	75	58

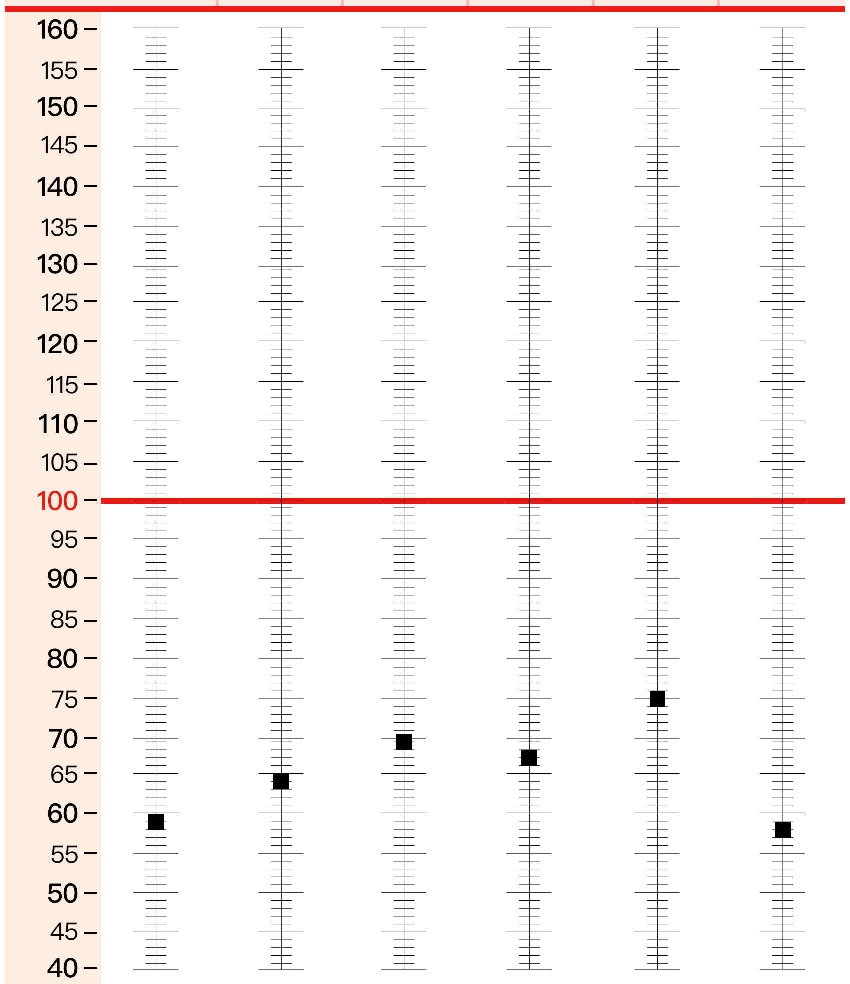

备注：

PEARSON
京美心测

《WPPSI-IV 中文版》经 NCS Pearson, Inc. 授权，由珠海市京美心理测量技术开发有限公司翻译，改编和印制。

版权所有，翻版必究。

主要分析页

指数水准 强项与弱项确定表

		指数分数		指数分数均值		分数差异	临界值	强项和弱项	基础率
与五个指数的平均数比	言语理解指数	59	−	66.80	=	-7.80	9.61		%
	视觉空间指数	64	−	66.80	=	-2.80	11.55		%
	流体推理指数	69	−	66.80	=	2.20	8.94		%
	工作记忆指数	67	−	66.80	=	0.20	9.98		%
	加工速度指数	75	−	66.80	=	8.20	12.66		%

比较选择

比较分数

指数分数均值	5个指数总分		指数分数均值
	334	÷5=	66.80

统计显著性水平

0.05

比较基础

全样本

		指数分数		总智商		分数差异	临界值	强项和弱项	基础率
与总智商比	言语理解指数	59	−	58	=	1	8.70		%
	视觉空间指数	64	−	58	=	6	11.83		%
	流体推理指数	69	−	58	=	11	9.83	强项	10 %
	工作记忆指数	67	−	58	=	9	10.96		%
	加工速度指数	75	−	58	=	17	13.06	强项	10 %

比较分数

总智商	总智商
	58

统计显著性水平

0.05

比较基础

全样本

确定指数水准的强项与弱项，请参照WPPSI-IV（CN）指导手册附表B. 1和 B. 2。

分测验水准 强项与弱项确定表

		分测验量表分数		分测验量表分数均值		分数差异	临界值	强项或弱项	基础率
与十个分测验量表分数均值比	常识	1	−	3.9	=	-2.9	3.08		%
	类同	3	−	3.9	=	-0.9	2.56		%
	积木	3	−	3.9	=	-0.9	3.22		%
	拼图	4	−	3.9	=	0.1	3.43		%
	矩阵推理	3	−	3.9	=	-0.9	2.47		%
	图画概念	6	−	3.9	=	2.1	2.54		%
	图片记忆	4	−	3.9	=	0.1	2.49		%
	动物家园	4	−	3.9	=	0.1	3.03		%
	找虫	4	−	3.9	=	0.1	3.29		%
	划消	7	−	3.9	=	3.1	3.84		%

比较选择

比较分数

分测验均值	10个指数分测验量表分数总和		分测验量表分数均值
	39	÷10=	3.9

统计显著性水平

0.05

10个指数分数分测验量表分数的分散度

6

确定分测验水准的强项与弱项，请参照WPPSI-IV（CN）指导手册附表B. 3和B. 4。

主要分析页（续）

分测验水准 强项与弱项确定表

与六个分测验量表分数均值比	分测验量表分数		分测验量表分数均值		分数差异	临界值	强项或弱项	基础率
常识	1	-	3.00	=	-2.00	2.92		%
类同	3	-	3.00	=	0.00	2.47		%
积木	3	-	3.00	=	0.00	3.05		%
拼图	4	-	3.00	=	1.00	3.87		%
矩阵推理	3	-	3.00	=	0.00	2.40		%
图画概念	6	-	3.00	=	3.00	2.88	强项	5-10%
图片记忆	4	-	3.00	=	1.00	2.42		%
动物家园	4	-	3.00	=	1.00	3.42		%
找虫	4	-	3.00	=	1.00	3.11		%
划消	7	-	3.00	=	4.00	4.32		%

比较选择

比较分数

	6个总智商分测验量表分数总和		分测验量表分数均值
分测验均值	18	÷ 6 =	3.00

统计显著性水准

0.05

6个指数分数分测验量表分数的分散度

3

确定分测验水准的强项与弱项，请参照WPPSI-IV（CN）指导手册附表B. 3和 B. 4。

差异比较表

指数水平	指数分数1		指数分数2		分数差异	临界值	显著性差异	基础率
言语理解 - 视觉空间	59	-	64	=	-5	12.81	否	%
言语理解 - 流体推理	59	-	69	=	-10	10.59	否	%
言语理解 - 工作记忆	59	-	67	=	-8	11.45	否	%
言语理解 - 加工速度	59	-	75	=	-16	13.78	是	15.6%
视觉空间 - 流体推理	64	-	69	=	-5	12.33	否	%
视觉空间 - 工作记忆	64	-	67	=	-3	13.07	否	%
视觉空间 - 加工速度	64	-	75	=	-11	15.16	否	%
流体推理 - 工作记忆	69	-	67	=	2	10.91	否	%
流体推理 - 加工速度	69	-	75	=	-6	13.34	否	%
工作记忆 - 加工速度	67	-	75	=	-8	14.03	否	%

比较选择

统计显著性水准

0.05

比较基础

全样本

分测验水平	量表分数1		量表分数2		分数差异	临界值	显著性差异	基础率
常识—类同	1	-	3	=	-2	2.97	否	%
积木—拼图	3	-	4	=	-1	3.51	否	%
矩阵推理—图画概念	3	-	6	=	-3	2.54	是	17.2%
图片记忆—动物家园	4	-	4	=	0	2.81	否	%
找虫—划消	4	-	7	=	-3	3.80	否	%

比较选择

统计显著性水准

0.05

比较指数分数之间或分测验分数之间的差异，请参照WPPSI-IV（CN）指导手册附表B. 5, B. 6, B. 7和 B. 8。

辅助分析页

量表分数总和

分测验/过程分数	量表分数				
积木		3	3		
常识			1		
矩阵推理		3	3		
找虫		4		4	
图片记忆		4		4	
类同			3		
图画概念		6	(6)		
划消		(7)		7	
动物家园		(4)		4	
拼图		(4)	(4)		
动物译码		(5)		(5)	
指认图片	2				
图片命名	2				
随机划消					9
有序划消					6
量表分数总和	4	20	10	19	
	语言接收	非言语	一般能力	认知效率	

辅助指数分数剖析图

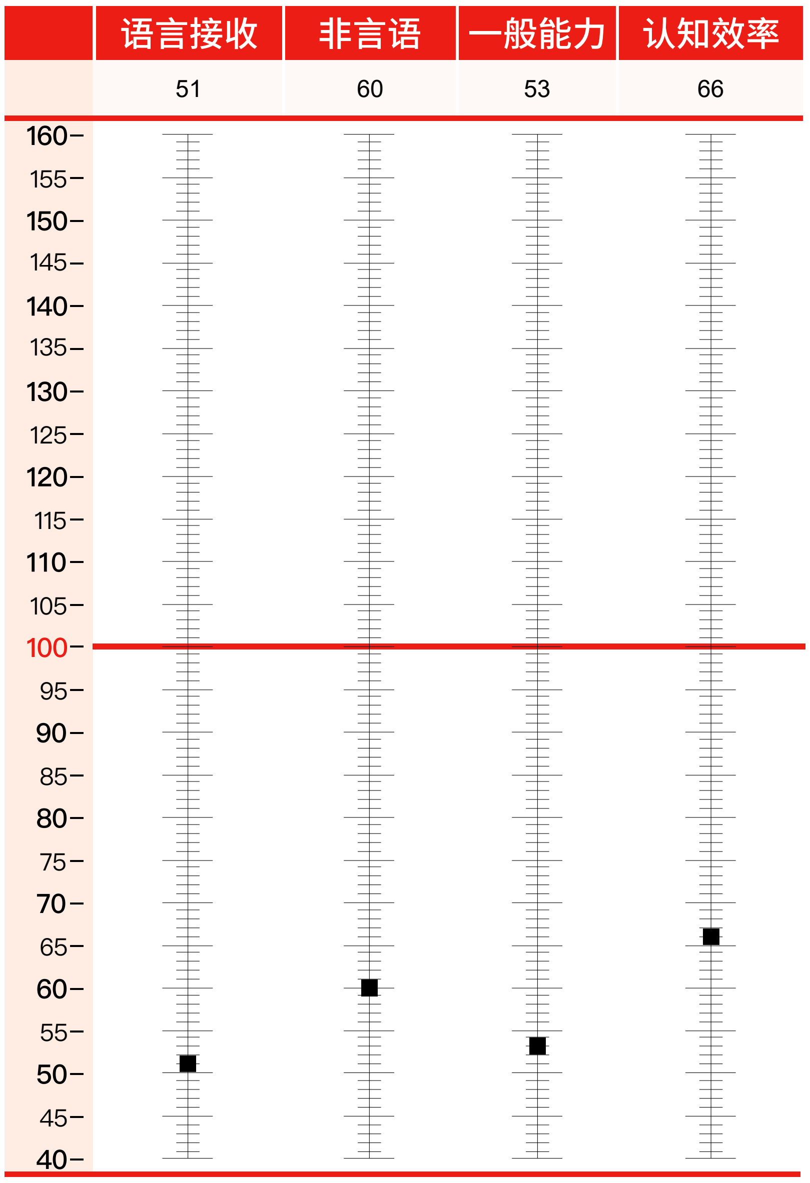

量表分数总和与辅助指数分数转换表

量 表	量表分数总和	辅助指数分数		百分等级	置信区间 95%
语言接收	4	VAI	51	0.1	47-63
非言语	20	NVI	60	0.4	56-68
一般能力	10	GAI	53	0.1	49-62
认知效率	19	CPI	66	1.0	61-76

差异比较表

	比 较	指数/量表分数1			指数/量表分数2		分数差异	临界值	显著性差异	基础率
指数水平	一般能力指数-总智商	GAI	53	-	FAIQ	58	= -5	3.90	是	13.0 %
指数水平	一般能力指数-认知效率指数	GAI	53	-	CPI	66	= -13	10.42	是	16.2 %
分测验水平	指认图片-图片命名	RV	2	-	PN	2	= 0	3.02	否	%
过程水平	随机划消-有序划消	CAR	9	-	CAS	6	= 3	4.65	否	%

比较选择
统计显著水平
0.05
比较基础
全样本

比较选择
统计显著水平
0.05
